# Systemic role of orexin A, substance P, bradykinin, and DABK in severe COVID-19 and 2.5-yr follow-ups: an observational study

**DOI:** 10.1016/j.bjao.2025.100415

**Published:** 2025-06-05

**Authors:** Ulrike Heinicke, Steven R. Talbot, Filippos Thanasis, Elisabeth H. Adam, Andreas von Knethen, Andrea U. Steinbicker, Sebastian Zinn, Kai Zacharowski, Armin N. Flinspach

**Affiliations:** 1 Department of Anaesthesiology, Intensive Care Medicine and Pain Therapy, Goethe University, University Hospital Frankfurt, Frankfurt am Main, Germany; 2 Institute for Laboratory Animal Science, Hannover Medical School, Hannover, Germany; 3 Fraunhofer – Institute for Translational Medicine and Pharmacology (ITMP), Frankfurt am Main, Germany; 4 Columbia University Medical Center, Department of Anesthesiology, New York, NY, USA

**Keywords:** acute respiratory distress syndrome, deep sedation, des-Arg^9^-bradykinin, intensive care unit, orexin A, post-acute COVID-19 syndrome, renin–angiotensin system, SARS-CoV-2

## Abstract

**Background:**

Orexin A regulates sleep–wake cycles, arousal, and energy homeostasis, linking it to the renin–angiotensin system and substance P. Dysfunction in these pathways occurs in acute and long-term COVID-19, including post-COVID syndrome.

**Methods:**

This observational study analysed plasma orexin A, substance P, bradykinin, and des-Arg^9^-bradykinin (DABK) in 78 ICU COVID-19 patients, 14 survivors of severe COVID-19 (2.5-yr follow-ups), and 14 healthy controls.

**Results:**

During acute COVID-19, bradykinin and substance P were significantly reduced, whereas DABK was elevated compared with healthy controls and 2.5-yr follow-ups. Orexin A concentration correlated with ICU survival (Cohen’s d=0.4), length of stay (LOS; r=–0.26, *P*=0.02), and sedation concentrations. Intriguingly, substance P plasma concentrations were elevated in 2.5-yr follow-ups. Plasma orexin A, substance P, and bradykinin increased with lower Richmond Agitation–Sedation Score (RASS): a combination of orexin A, substance P, and bradykinin concentrations at RASS –3 to –5 distinguished survivors from non-survivors of COVID-19 when categorised by age.

**Conclusions:**

Changes in the bradykinin axis, affecting substance P and orexin A signalling, are associated with severe COVID-19, ICU LOS, and survival. Elevated substance P concentrations in the 2.5-yr follow-up cohort may be associated with physical, cognitive, and neuropsychological impairments commonly seen in post-ICU syndrome and post-COVID syndrome. The predictive values of orexin A, substance P, bradykinin, and DABK and the complex interplay between the renin–angiotensin system and the orexinergic system in severe, critical illnesses or viral diseases will be investigated in future studies.

Orexin A, also known as hypocretin-1, is a neuropeptide produced in the hypothalamus that regulates wakefulness, appetite, and energy homeostasis in the brain.^1^^,^^2^ It interacts with orexin receptors (OX_1_R and OX_2_R), activating different downstream signalling pathways and exerting various physiological functions.^3^^,^^4^ Loss of orexin neurons or altered orexin A plasma concentrations have been described in several neurological diseases.^5^, ^6^, ^7^, ^8^, ^9^ Thereby, a loss in orexin signalling results in narcolepsy in mice, dogs, and humans.^5^^,^^10^^,^^11^ Hypoxic arousal events in patients with sleep apnoea have also been linked to increased concentrations of orexin A.^12^ Orexin antagonists represent a novel drug class that has been shown to stabilise slow-wave sleep. Besides the brain, orexin receptors have also been detected, for example, in the kidneys, adrenal glands, gonads, pancreas, lungs, and intestine, indicating that their scope of action is much broader. Orexin A influences not only energy homeostasis, sleep–awake behaviour, nociception reward seeking, and food and drug addiction, but also reproduction, cardiovascular, and adrenal function.^13^, ^14^, ^15^ In addition, recent research suggests that orexin A may also play a significant role in the modulation of the immune response and inflammatory processes, which are critically implicated in the pathogenesis of severe COVID-19.^16^, ^17^, ^18^, ^19^

Severe acute respiratory syndrome coronavirus-2 (SARS-CoV-2), causing COVID-19, emerged as a global health crisis in late 2019 and has since led to significant morbidity and mortality worldwide. Currently, there is resurgence of COVID-19 worldwide. Since July–August 2024, there has been a noticeable increase in COVID-19 cases, with a significant increase in hospitalisations and ICU admissions in many regions, including the Americas, Europe, and the Western Pacific.^20^ Among the affected populations, critically ill patients who require ICU admission represent a group with particularly severe manifestations of the disease. These patients usually suffer from COVID-19-induced acute respiratory distress syndrome (CARDS) and often develop sepsis and multiorgan failure during their ICU stay, requiring advanced supportive measures such as mechanical ventilation or extracorporeal membrane oxygenation (ECMO).^21^^,^^22^ In addition, increased doses of hypnotics were used to treat patients in order to achieve the desired Richmond Agitation–Sedation Score (RASS).^23^ We, and numerous others, highlighted the need for drug combinations and increased doses of anaesthetic agents to achieve sufficient respiratory synchrony with ventilatory devices with a possible cost of neuronal impairment.^24^, ^25^, ^26^ A proinflammatory immune response might contribute to both the observed hypermetabolism and sensitivity to arousal stimuli.^27^

We reported previously that des-Arg^9^ bradykinin (DABK) and not bradykinin is significantly elevated in critically ill COVID-19 patients and is associated with disease severity, inflammatory markers, and survival.^28^ Furthermore, we identified that bradykinin and substance P, both inactivated by the angiotensin-converting enzyme (ACE), are diminished in critically ill COVID-19 patients. The interplay between bradykinin and substance P and their cognate signalling pathways could influence the activity of orexin neurons through direct interaction or indirect effects via other neurochemical systems.^29^ Studies in rodents have shown that substance P can affect the excitability of hypothalamic neurons, thereby indicating that substance P can potentially also affect orexin A-producing neurons.^30^, ^31^, ^32^ Research has also shown that inflammatory processes and pain responses, in which substance P plays a role, can alter the activity of orexin neurons.^16^^,^^33^, ^34^, ^35^ However, studies directly analysing the potential role of orexin-A in COVID-19 or post-COVID syndrome are still lacking.

Therefore, the current study aimed to identify the clinical and physiological role of orexin A in severe COVID-19 patients and 2.5-yr follow-ups, compared with healthy controls. Furthermore, we want to understand the interplay between orexin A, substance P, bradykinin, and DABK and the immune–metabolic setting of those patients, and also its effect on the unusual level of sedation in COVID-19 patients.

## Methods

### Ethics approval and consent to participate

The study was performed in compliance with the Declaration of Helsinki.^36^ Approval from the local ethics committee (Ethik-Kommission des Fachbereichs Medizin der Goethe-Universität c/o Universitätsklinikum) was obtained before the study started (reference #20-643 [07 April 2020], #20-982 [07 January 2021], #2022-701 [08 August 2022]), and a waiver regarding the requirement of written informed consent from COVID-19 patients was authorised by the local ethics committee (Ethik-Kommission des Fachbereichs Medizin der Goethe-Universität c/o Universitätsklinikum). All participants in the control group provided written informed consent.

### Study cohorts

**Fig 1:**
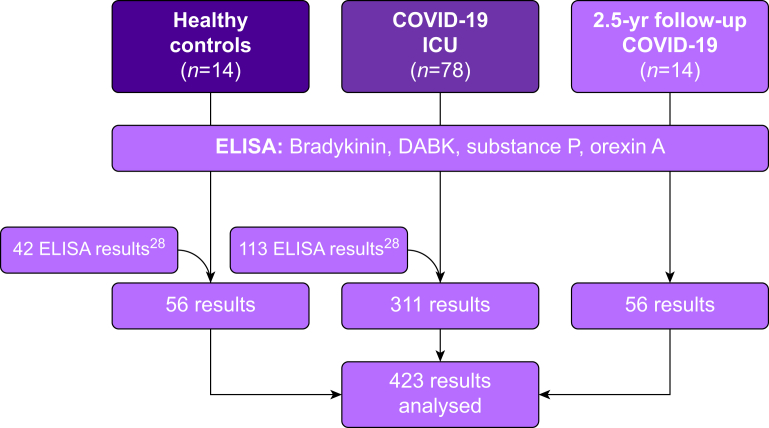
**Flow chart.** This figure illustrates the study design. A total of 423 enzyme-linked immunosorbent assay (ELISA) results were analysed. Some ELISA results from healthy controls (*n*=42) and from COVID-19 intensive care patients (*n*=113) have already been published previously.^28^DABK, Des-Arg^9^-bradykinin; ELISA, enzyme-linked immunosorbent assay; ICU, Intensive Care Unit

This is a retrospective single-centre cohort study of 78 COVID-19 patients (age ≥18 yr) admitted to the ICU of the University Hospital Frankfurt am Main, Germany, between June 2020 and November 2021 (first to fourth wave; prevalent wild-type SARS-CoV-2). Survivors of COVID-19 or their relatives were contacted 2.5 yr (plus or minus 6 months) after discharge to provide information about their health status and offer follow-up appointments. In this context, blood samples were drawn from 14 survivors of COVID-19 (age >18 yr), presenting to a 2.5-yr (plus or minus 6 months) follow-up (follow-up cohort). Patient care and study conduct complied with good clinical practice and current guidelines. Blood samples were obtained daily between 8:00 and 11:00 a.m. during the ICU stay. Inflammatory markers, including C-reactive protein (CRP), interleukin 6 (IL-6), procalcitonin (PCT), lactate dehydrogenase (LDH), and peripheral leucocyte count were measured daily at 4:00 a.m. by the central laboratory department and compared with the central laboratory’s threshold concentrations. Control samples were drawn from 14 healthy donors (age ≥18yr). The enzyme-linked immunosorbent assay (ELISA) results from 42 healthy controls and 113 COVID-19 ICU patients have been previously published elsewhere (Fig. 1, Table 1).^28^

**Table 1:** Patient characteristics of the COVID-19 cohort and 2.5-yr follow-up cohort. Data are presented as *n* (%) for categorical variables or median (inter-quartile range) for continuous variables. The patients’ laboratory variables are reported as the respective median of the concentrations obtained during ICU stay. CRP, C-reactive protein; IL-6, interleukin 6; LDH, lactate dehydrogenase; PCT, procalcitonin.

Total	COVID-19	Follow-up
	78 (100)	14 (100)
Sex (female)	20 (25.6)	4 (28.6)
Age (yr)	61 (49–70.3)	60.5 (53.5–73.3)
BMI (kg m^–2^)	30 (26.1–33)	30.7 (27.1–33.2)
ICU stay (days)	15 (8–26)	–
Death	40 (51.3)	–
Comorbidity		
Cardiovascular disease	44 (56.4)	10 (71.4)
Respiratory disease	14 (17.9)	4 (28.8)
Diabetes mellitus	23 (29.5)	3 (21.4)
Obesity	17 (21.8)	8 (57.1)
Narcolepsy	0 (0)	0 (0)
Schizophrenia	0 (0)	0 (0)
Depression	3 (3.8)	3 (21.4)
Bipolar disorder	0 (0)	0 (0)
Laboratory variables		
CRP (mg dl^–1^)	11.9 (4.2–20.9)	0.13 (0.1–0.4)
Leucocyte count (10⁹/L)	9.5 (7.4–13.8)	7.5 (5.8–8.6)
IL-6 (pg ml^–1^)	114.0 (22.7–465.5)	–
PCT (ng ml^–1^)	0.43 (0.14–1.51)	–
LDH (U L^–1^)	480 (348.0–615.0)	200 (185.3–231.5)
Creatinine (mg dl^–1^)	0.95 (0.68–1.34)	0.92 (0.77–1.13)

### Plasma preparation and measurement

Whole blood samples were drawn into citrate tubes (Citrate 3, 13%; SARSTEDT S-Monovette, Nümbrecht, Germany) and subsequently centrifuged for 10 min at 2000×*g* before being stored at –80°C until further processing. ELISAs (bradykinin – Ab136936, Abcam PLC, Cambridge, UK; DABK [DA9BK] – MBS109439, MyBioSource Inc., San Diego, CA, USA; substance P – ADI-900-018, Enzo Life Sciences Inc., Farmingdale, NY, USA; orexin A – abx252876, Abbexa, Cambridge, UK) were used to determine the plasma concentration of bradykinin, DABK, substance P, and orexin A.

### Statistical analysis

Data are presented as *n* (%) for categorical variables or median (inter-quartile range [IQR]) for continuous variables. The laboratory test results are reported as the respective median during ICU stay. Statistical analyses were performed with GraphPad Prism 10.0 (GraphPad Software, San Diego, USA). Quantitative variables of non-Gaussian distributed data were shown as medians with IQRs (P25–P75%). Nominal data were expressed as counts and percentages. The Shapiro–Wilk test was used to test data against the hypothesis of normal distribution. Single-group comparisons of continuous data were analysed with Student’s *t*-test (parametric data) or Mann–Whitney *U* test (non-parametric data). When data did not conform to a normal distribution, the Kruskal–Wallis test with Dunn’s *post hoc* test was used to compare more than two groups. Fisher’s exact test was used for categorical data. Principal component analysis (PCA) was used to investigate the relevance of the measurements and their correlations. Effect sizes were calculated using Cohen’s d. A Cohen’s d value of 0.2 indicated a small effect, 0.5 a medium effect, and 0.8 a large effect.^37^ Trend analyses were performed with linear regressions, in which the dependent variables (e.g. orexin A) were modelled as functions of the independent variable (e.g. ICU stay). Heat maps were constructed from Spearman correlations. Pairs of samples were tested for association and reported as Spearman’s r and the corresponding *P*-value. A *P*-value ≤0.05 was considered statistically significant in all statistical tests and was further reported for the following concentrations: ∗*P*≤0.05, ∗∗*P*≤0.01, ∗∗∗*P*≤0.001. Differences within bradykinin, substance P, and orexin A were assessed as a function of the number of anaesthetic drugs and RASS (–3 to –5) using an analysis of variance (anova). As no statistically significant differences were observed, no *post hoc* analysis was performed.

## Results

### Participant characteristics

In this study, 78 COVID-19 patients were admitted to the ICU (COVID-19 ICU cohort; Table 1, Fig. 1). Plasma samples from patients who survived COVID-19 after treatment in an ICU (*n*=14) and presented 2 yr later for follow-up examination (2.5-yr follow-up COVID-19 cohort; Table 1, Fig. 1), and also plasma samples (*n*=14) from healthy controls (age ≥18 yr) were obtained (Fig. 1).

Of the COVID-19 cohort, 38 (48.7%) patients survived and 40 (51.3%) did not survive COVID-19. The median length of ICU stay of the COVID-19 cohort was 15 (IQR 8–26) days. We found a significantly increased rate of obesity (*P*=0.018) and depression (*P*=0.043) in the 2.5-yr follow-up patients compared with the COVID-19 cohort (Table 1). The median concentrations of CRP (*P*≤0.0001) and LDH (*P*≤0.0001), and the median leucocyte count (*P*=0.0066) were significantly higher in the COVID-19 ICU cohort than in the survivors of severe COVID-19 (2.5-yr follow-up cohort). The IL-6 and PCT concentrations were increased in the COVID-19 ICU cohort compared with the central laboratory threshold but were not analysed in the 2.5-yr follow-up cohort.

### Factors potentially affecting orexin A concentrations in the COVID-19 cohort

To avoid circadian fluctuations of orexin A levels in our experimental setup, plasma samples were always taken simultaneously (∼8-11 a.m.). To further exclude possible effects of sex, age, BMI, or glucose, we analysed if there were any differences in orexin A plasma levels of our COVID-19 cohort (Supplementary Fig. S1A–D). Interestingly, the distribution of orexin A plasma concentrations from COVID-19 patients resembled an hourglass, with half of the values above and half below the control threshold. There were no statistical differences in orexin A plasma concentrations between COVID-19 patients stratified by sex, age, BMI, or glucose concentration (Supplementary Fig. S1A–D).

There were no significant differences in plasma orexin A concentrations of COVID-19 patients suffering from cardiovascular disease, respiratory disease, or obesity (Supplementary Fig. S2A–C). Notably, COVID-19 patients with diabetes mellitus had significantly lower plasma orexin A concentrations than those without diabetes (*P*=0.049; Table 2; Supplementary Fig. S2D). However, insulin given to COVID-19 patients with diabetes mellitus did not affect orexin A plasma concentrations (Supplementary Fig. S2E).

**Table 2:** Absolute differences and effect sizes of orexin A. Mean values and the absolute difference in mean values between males/females, COVID-19 patients with or without cardiovascular disease, COVID-19 patients with or without respiratory diseases, survivors/non-survivors, and the effect size (Cohen’s d) for orexin A. A Cohen’s d of 0.2 indicates a small effect, 0.5 a medium effect, and 0.8 a large effect.^37^

	No. of patients	Mean value (pg ml^–1^)	Absolute difference in mean value (pg ml^–1^)	Cohen’s d
Male	58	1971	153	0.24
Female	20	2124		
Without cardiovascular disease	34	2161	266	0.43
With cardiovascular disease	44	1895		
Without respiratory disease	64	2041	168	0.27
With respiratory disease	14	1873		
Survivors	38	2138	248	0.40
Non-survivors	40	1890		

The absolute differences in mean orexin A concentration between males and females was 153 pg ml^–1^, between COVID-19 patients with or without cardiovascular disease was 266 pg ml^–1^, and between COVID-19 patients with or without respiratory disease was 168 pg ml^–1^ (Table 2). Taking the pooled standard deviation into account, this results in a Cohen’s d of 0.24 (males *vs* females), 0.43 (COVID-19 patients with or without cardiovascular disease), and 0.27 (COVID-19 patients with or without respiratory disease) for orexin A, indicating a small to medium effect (Table 2).

### Plasma concentrations of bradykinin, DABK, substance P, and orexin A normalised 2 yr after severe COVID-19

**Fig 2:**
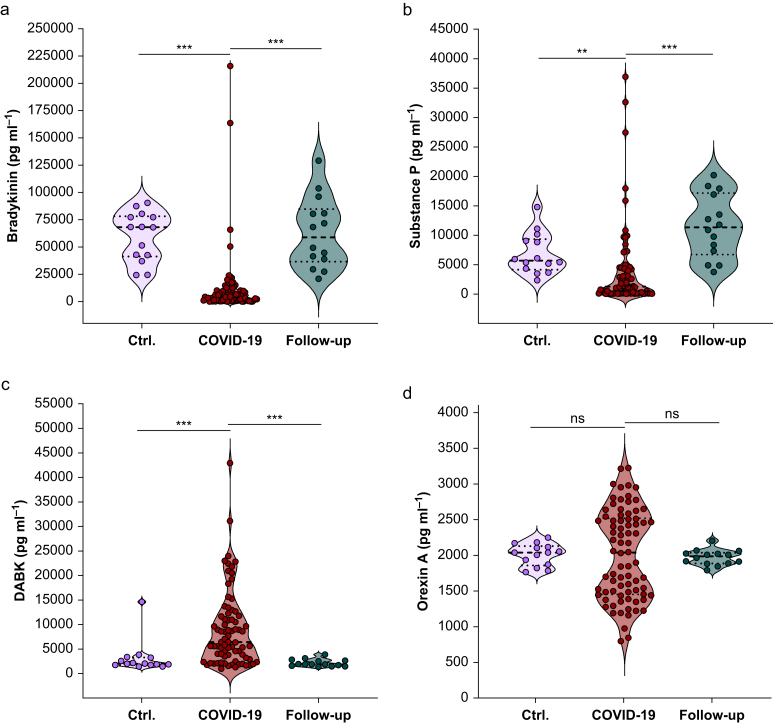
Bradykinin, substance P, DABK, and orexin A plasma concentrations differ between acute intensive care COVID-19 patients and 2.5-yr follow-ups. Violin plots of plasma bradykinin (a), substance P (b), DABK (c), and orexin A (d) are shown. In total, values of 14 healthy controls (Ctrl), 78 acute COVID-19 intensive care patients, and 14 survivors of severe COVID-19 presenting to a 2.5-yr follow-up examination (follow-up), independently of time, are depicted. Statistical differences between the groups were assessed using the Kruskal–Wallis test; ∗∗∗*P*<0.001. DABK, des-Arg^9^-bradykinin.

As previously shown, plasma concentrations of bradykinin and substance P from our COVID-19 ICU cohort were significantly lower than those of healthy controls (Fig. 2a and b).^28^ In contrast, plasma concentrations of DABK were significantly higher in COVID-19 patients than in healthy controls (Fig. 2c).^28^ There was no difference in orexin A plasma concentration between COVID-19 patients and healthy controls (Fig. 2d). The extraordinary sample distribution of orexin A in our COVID-19 cohort was not dependent on sex, age, BMI, glucose, insulin, cardiovascular disease, obesity, or diabetes mellitus (Supplementary Figs 1 and 2).

Former COVID-19 intensive care patients who presented 2 yr after ICU discharge to a follow-up examination displayed normal plasma concentrations of DABK and orexin A (Fig. 2c and d). In contrast, plasma substance P concentrations were higher in 2.5-year follow-up patients than in healthy controls but not statistically different. However, the absolute difference in mean values between follow-ups and healthy controls for substance P was 4687 pg ml^–1^ (Table 3). Considering the pooled standard deviation, this resulted in a Cohen’s d of –1.03 for substance P, indicating a large effect (Table 3).

**Table 3:** Absolute differences and effect sizes of substance P. Mean values, the absolute difference in mean values between healthy controls and 2.5-yr follow-up cohort, and the effect size (Cohen’s d) for substance P. A Cohen’s d of 0.2 indicates a small effect, 0.5 a medium effect, and 0.8 a large effect.^37^

	Cohort	Mean value (pg ml^–1^)	Absolute difference in mean value (pg ml^–1^)	Cohen’s d
Substance P	Healthy controls	6826	4687	–1.03
	2.5-yr follow-up	11 513		

### Clinical association of orexin A with worsening of the disease in severe COVID-19

**Fig 3:**
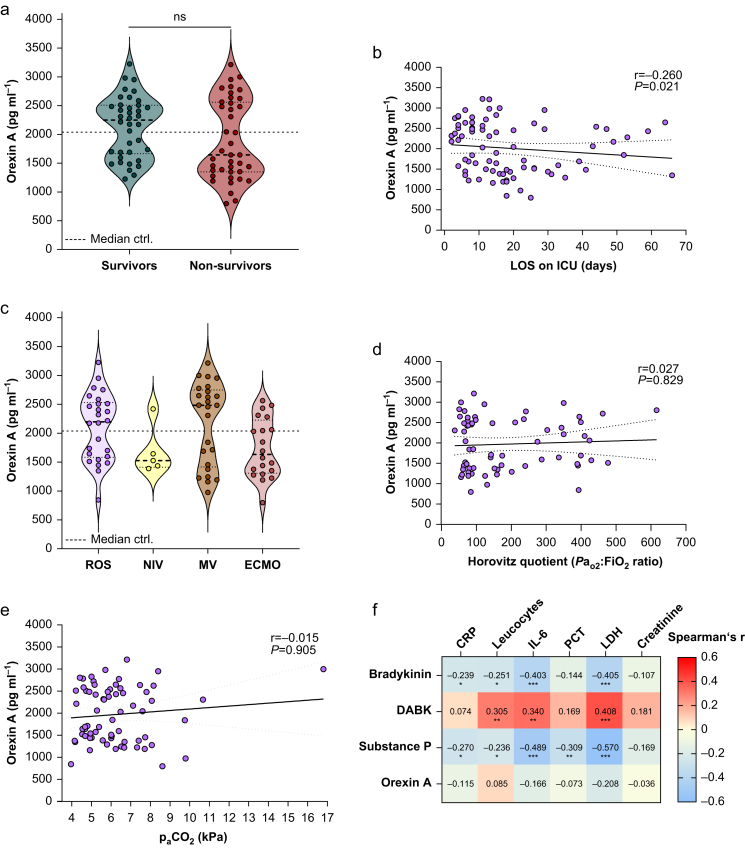
Association of bradykinin, substance P, DABK, and orexin A with disease severity. Violin plots of plasma orexin A for survivors (*n*=38) and non-survivors (*n*=40) of COVID-19 patients (a), independently of time, are shown. Statistical differences between survivors and non-survivors of COVID-19 were assessed using the Mann–Whitney *U* test. Plasma orexin A concentrations were plotted against ICU stay (b). Statistical differences were evaluated using Spearman’s correlation test. Trend analysis was performed with linear regression. Mean and errors (dotted lines) are shown. Violin plots of plasma orexin A for different ventilation modes, independently of time, are shown (c). Statistical differences were assessed using the Kruskal–Wallis test. Plasma orexin A concentrations were plotted against the ratio of partial pressure of arterial oxygen and fraction of inspired oxygen ($Pa_{o_{2}}$:FiO_2_) ratio (d). Statistical differences were evaluated using Spearman’s correlation test. Trend analysis was performed with linear regression. Mean and errors (dotted lines) are shown. Heat map representing Spearman’s colour-coded correlation coefficients between the values of bradykinin, substance P, DABK, or orexin A and inflammatory markers are shown (e). Statistical differences were assessed using Spearman’s correlation test; ∗*P*<0.05, ∗∗*P*<0.01, ∗∗∗*P*<0.001. The median of 14 healthy controls is depicted as a dotted line in a and c. CRP, C-reactive protein; DABK, des-Arg^9^-bradykinin; ECMO, extracorporeal membrane oxygenation; IL-6, interleukin 6; LDH, lactate dehydrogenase; NIV, noninvasive ventilation; PCT, procalcitonin.

The median plasma orexin A concentrations were lower in non-survivors than in survivors of COVID-19, but statistical significance was not demonstrated because of the distribution of orexin A concentrations (Fig. 3a). However, the absolute difference in mean values between survivors and non-survivors was 248 pg ml^–1^ for orexin A (Table 2). Considering the pooled standard deviation, this resulted in a Cohen’s d of 0.40 for orexin A, indicating a medium effect (Table 2).

Orexin A concentrations were negatively and significantly related to the length of stay (LOS) of COVID-19 patients in the ICU (Fig. 3b). There were no significant differences in orexin A plasma concentrations in COVID-19 patients depending on their requirement for oxygen supplementation (ROS; *n*=26), need for noninvasive ventilation (NIV; *n*=5), mechanical ventilation (MV; *n*=25), and the implementation of ECMO (*n*=20; Fig. 3c). However, the absolute differences in mean values between COVID-19 patients on ROS, NIV, MV, or ECMO and healthy controls were 99, 328, 170, and 272 pg ml^–1^ for orexin A, respectively (Table 4). Considering the pooled standard deviation, this resulted in a Cohen’s d of 0.21 for ROS, –1.34 for NIV, 0.29 for MV, and –0.67 for ECMO, indicating small to large effects (Table 4).

**Table 4:** Absolute differences and effect sizes of orexin A. Mean values and the absolute difference in mean values between requirement for oxygen supplementation (ROS), noninvasive ventilation (NIV), mechanical ventilation (MV), extracorporeal membrane oxygenation (ECMO), and healthy controls and the effect size (Cohen’s d) for orexin A. A Cohen’s d of 0.2 indicates a small effect, 0.5 a medium effect, and 0.8 a large effect.^37^

	No. of patients	Mean value (pg ml^–1^)	Absolute difference in mean value (pg ml^–1^)	Cohen’s d
ROS	26	2109	99	0.21
NIV	5	1682	328	–1.34
MV	25	2180	170	0.29
ECMO	20	1738	272	–0.67
Healthy controls	20	2010	–	–

To further reveal if there was a possible association between ventilation and orexin A, we next analysed the ratio of partial pressure of oxygen in blood and the fraction of inspired oxygen ($Pa_{o_{2}}$:FiO_2_), also known as Horovitz quotient, as a clinical marker to assess lung function and evaluate lung damage (Fig. 3d). However, unlike bradykinin, substance P, and DABK, orexin A did not correlate with the $Pa_{o_{2}}$:FiO_2_ ratio (Fig. 3d; Supplementary Fig. S3A–C). As orexin neurons serve as primary chemoreceptors and are depolarised by elevated carbon dioxide concentrations, we also analysed whether carbon dioxide concentrations are associated with orexin A (Fig. 3e). Unlike bradykinin and substance P, DABK and orexin were unrelated to carbon dioxide (Fig. 3e; Supplementary Fig. S3D–F).

In line with our previously published results, we observed that bradykinin and substance P were negatively and significantly related to CRP, leucocyte count, IL-6, PCT (only substance P), and LDH.^28^ Furthermore, DABK showed a positive and significant relation to leucocyte count, IL-6, and LDH (Fig. 3e).^28^ However, no significant associations between plasma concentrations of orexin A and these inflammatory markers were detected (Fig. 3f).

### Association of orexin A with disease progression and RASS in COVID-19 patients

**Fig 4:**
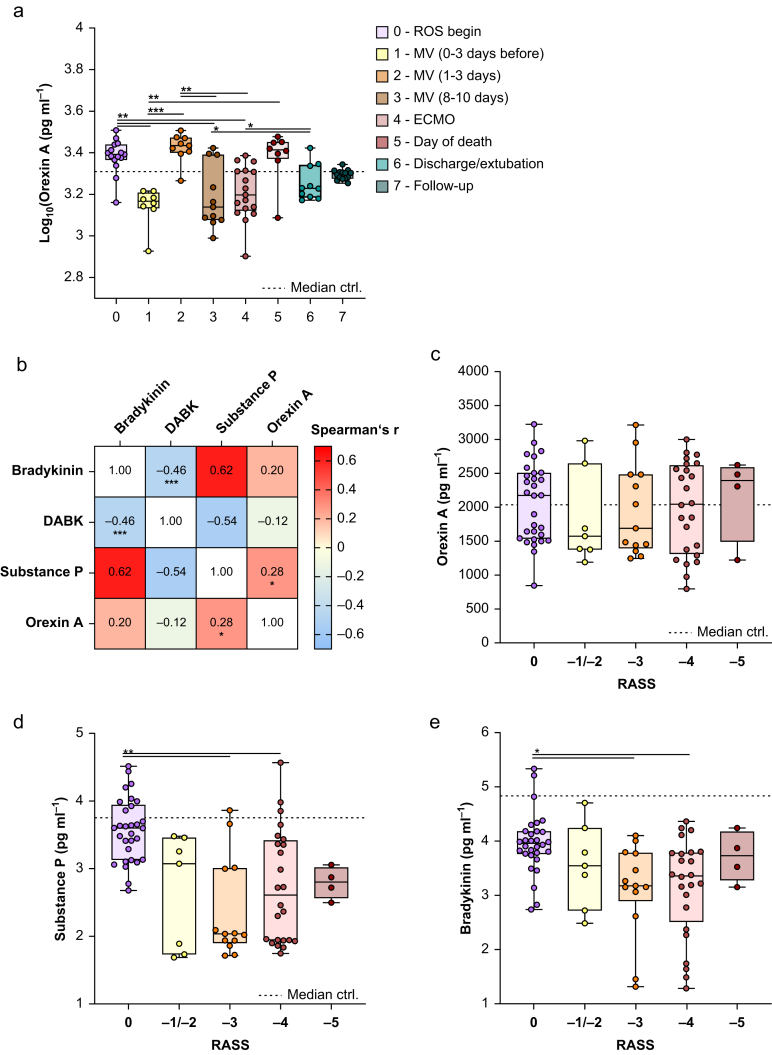
Association of orexin A with COVID-19 progression and Richmond Agitation–Sedation Score (RASS). Box plots of log_10_ plasma orexin A concentrations depending on disease progression are shown (a). Patients of the COVID-19 cohort and 2.5-yr follow-up cohort were divided into eight groups resembling the clinical course of COVID-19. Patients requiring oxygen supplementation (ROS) at the beginning of the disease were divided into Group 0 (*n*=15), patients 0–3 days before connection to mechanical ventilation (MV) into Group 1 (*n*=8), patients 1–3 days after connection to MV into Group 2 (*n*=9), patients 8–10 days after connection to MV into Group 3 (*n*=11), patients with implemented extracorporeal membrane oxygenation (ECMO) into Group 4 (*n*=17), patients where plasma samples were collected on the day of death into Group 5 (*n*=8), patients where plasma samples were collected on the day of tracheal extubation or discharge into Group 6 (*n*=10), and patients with 2.5-yr follow-up of COVID-19 into Group 7 (*n*=14). Heatmap representing Spearman’s colour-coded correlation coefficients between the values of bradykinin, substance P, DABK, and orexin A (b). Statistical differences were assessed using Spearman’s correlation test; ∗*P*˂0.05, ∗∗∗*P*˂0.001. Box plots of plasma concentrations depending on RASS are shown (c–e). The median of 14 healthy controls is depicted as a dotted line in a, c, d, e. Statistical differences were assessed using the Kruskal–Wallis test; ∗*P*<0.05, ∗∗*P*<0.01, ∗∗∗*P*<0.001.

We divided the COVID-19 cohort and 2.5-yr follow-up cohort into eight groups resembling the clinical course of COVID-19 (Fig. 4a, Groups 0–7). Patients who received ROS at the beginning of the disease (Fig. 4a, Group 0, *n*=15) had significantly higher orexin A plasma concentrations than patients directly before MV (Group 1, *n*=8), patients 8–10 days of MV (Group 3, *n*=11), and patients upon ECMO (Group 4, *n*=17). In addition, patients directly before MV (Group 1, *n*=8) had significantly lower orexin A plasma concentrations than patients 1–3 days after MV (Group 2, *n*=9) and patients on the day of death (Group 5, *n*=8). Furthermore, patients after 1–3 days of MV (Group 2, *n*=9) had significantly higher orexin A plasma concentrations than patients after 8–10 days of MV (Group 3, *n*=11) and patients upon ECMO (Group 4, *n*=17). Notably, patients after 8–10 days of MV (Group 3, *n*=11) had significantly lower orexin A plasma concentrations than patients on the day of discharge/tracheal extubation (Group 6, *n*=14).

Interestingly, the changes in orexin A plasma concentrations observed upon MV (Fig. 3c) could be traced back to the onset of MV, as patients 1–3 days after starting MV (Fig. 4a, Group 2) had significantly higher orexin A plasma concentrations than healthy controls, whereas patients after 8–10 days of MV (Group 3, *n*=11) had significantly lower orexin A plasma concentrations than healthy controls (Fig. 4a). Notably, the median orexin A concentration was lowest after 8–10 days of MV and then strongly increased with disease worsening to above the control value (Fig. 4a, Groups 4 and 5). Thereby, orexin A somehow acted similarly to bradykinin and substance P and contrary to DABK (Supplementary Fig. S4A–D). Accordingly, a correlation analysis between bradykinin, DABK, substance P, and orexin A plasma concentrations of COVID-19 patients revealed that orexin A was positively associated with substance P and bradykinin (Fig. 4b). In addition, bradykinin was positively and significantly related to substance P, and negatively and significantly related to DABK. Accordingly, DABK showed a negative and significant relation to bradykinin and substance P.

We next examined if the varying plasma orexin A concentrations upon disease worsening and ventilation were related to RASS (Fig. 4c). Notably, plasma levels of orexin A in the RASS = 0 group showed a broad distribution, ranging above and below the control threshold. This likely reflects the heterogeneity of patients at different stages of COVID-19 progression. Furthermore, as most of our COVID-19 patients were severely sick and required deep sedation, there were only a few patients in the RASS group –1/–2. Intriguingly, plasma orexin A concentrations were the lowest with RASS –1/–2 and then strongly increased with RASS –3 to –5, even above the control values (Fig. 4c). This pattern in plasma concentrations was also seen for bradykinin and substance P but not for DABK, where plasma concentrations decreased upon RASS –3 to –5 (Fig. 4d and e; Supplementary Fig. S4E).

### Associations between bradykinin, substance P, and orexin A in COVID-19 patients with RASS –3 to –5

It has been described before that anaesthetics can reduce the efficacy of orexin signalling through its receptors.^38^ As severe COVID-19 patients received high levels of sedation in a prone position and mechanical ventilation, we next analysed whether anaesthetics had a combined effect on plasma bradykinin, substance P, or orexin A levels in COVID-19 patients with RASS -3 to -5 (Supplementary Fig. S5). We found no statistically significant effect of the number of anaesthetic drugs on plasma bradykinin, substance P, or orexin A concentrations in COVID-19 patients with RASS –3 to –5 (Supplementary Fig. S5), but the variability was high with only a few patients who received one to two anaesthetic drugs.

**Fig 5:**
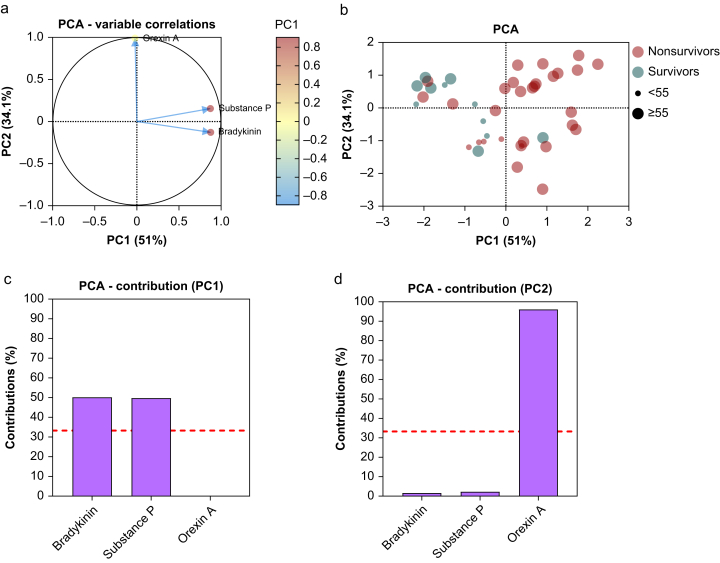
Principal component analysis (PCA) of bradykinin, substance P, and orexin A plasma concentrations in COVID-19 ICU patients with Richmond Agitation–Sedation Score (RASS) –3 to –5. PCA variable correlations plot of bradykinin, substance P, and orexin A plasma concentrations (a). With two dimensions, 85.1% of the variance is expressed. PCA scatter plot of the four groups (green, survivors of COVID-19; red, non-survivors of COVID-19; small circles, ≤55 yr of age; big circles, ≥55 yr of age) with plasma concentrations of all three markers (b). Bar plot of the variables’ (bradykinin, substance P, and orexin A) contribution to dimension 1 (Dim1) in percentage (c). Bar plot of the variables’ (bradykinin, substance P, and orexin A) contribution to dimension 2 (Dim2) in percentage (d). The red dashed line indicates the uniform distribution of all contributing variables (33%).

In line with our previous results, in patients with RASS –3 to –5, PCA revealed a positive association between bradykinin and substance P (Fig. 5a).^28^ Furthermore, combined measurements of orexin A, substance P, and bradykinin segregated non-survivors from survivors of COVID-19 in a PCA scatterplot using the first two principal components (Fig. 5b). This effect was even more pronounced in patients aged ≥55 yr. COVID-19 patients aged ≥55 yr had a lower BMI (29.4 *vs* 32.7), a higher rate of non-survivors (62% *vs* 28%), a significantly increased rate of cardiovascular (*P*=0.0156) and respiratory (*P*=0.0297) disease, and significantly higher concentrations of IL-6 (*P*=0.0314) than COVID-19 patients aged ≤55 yr (Table 5). The contributions of bradykinin, substance P, and orexin A to principal components 1 and 2 are depicted in Figure 5c and d.

**Table 5:** Patient characteristics of COVID-19 patients aged ≤55 yr and ≥55 yr. Data are presented as *n* (%) for categorical variables or median (inter-quartile range) for continuous variables. The patients’ laboratory variables are reported as the respective median of the concentrations obtained during ICU stay. *P*-values comparing COVID-19 and follow-ups were calculated with a *t*-test, Mann–Whitney *U* test, or Fisher’s exact test. CRP, C-reactive protein; DABK, des-Arg^9^-bradykinin; IL-6, interleukin 6; LDH, lactate dehydrogenase; PCT, procalcitonin.

Total	≤55 yr	≥55 yr	*P*-value
	25 (100)	53 (100)	
Sex (female)	10 (40)	10 (18.9)	0.056
Age (yr)	44 (34–49)	67 (60–73.5)	≤0.001
BMI (kg m^–2^)	32.7 (25.7–37.6)	29.4 (26.2–32.1)	0.024
ICU stay (days)	13 (6–21.5)	15 (8.5–26)	0.333
Death	7 (28)	33 (62.3)	0.007
Horovitz	108.0 (74.8–398.0)	136 (75–240.5)	0.504
Comorbidity			
Cardiovascular disease	9 (36)	35 (66)	0.016
Respiratory disease	1 (4)	13 (24.5)	0.030
Diabetes mellitus	11 (44)	12 (22.6)	0.066
Obesity	6 (24)	11 (20.8)	0.774
Laboratory variables			
Glucose	157 (141–185)	161 (131–192)	0.774
CRP (mg dl^–1^)	8.78 (1.4–19.8)	11.04 (6.4–23.7)	0.123
Leucocyte counts (nl^–1^)	8.59 (6.9–13.4)	9.56 (7.68–14.00)	0.320
IL-6 (pg ml^–1^)	50.1 (9.3–247.5)	137.0 (32.8–711.0)	0.031
PCT (ng ml^–1^)	0.41 (0.14–1.31)	0.46 (0.15–1.71)	0.454
LDH (U L^–1^)	481.5 (332.3–638)	480 (357.5–609.5)	0.676
Creatinine (mg dl^–1^)	0.76 (0.59–1.38)	1.00 (0.69–1.32)	0.246
Bradykinin (pg ml^–1^)	5483 (1467–9092)	5934 (1418–12 803)	0.807
DABK (pg ml^–1^)	9609 (2168–18 064)	5609 (3586–9855)	0.184
Substance P (pg ml^–1^)	1236 (82.33–3696)	1270 (301.6–4263)	0.364
Orexin A (pg ml^–1^)	2061 (1462–2488)	2032 (1451–2553)	0.992

## Discussion

To our knowledge, this study is the first to analyse plasma orexin A concentrations in patients with severe COVID-19 and 2.5-yr follow-ups. We demonstrated that orexin A plasma concentrations in COVID-19 patients were strikingly aberrant and that patients who recovered from severe COVID-19 and were discharged from the ICU exhibited elevated substance P plasma concentrations in a 2.5-yr follow-up. Furthermore, plasma orexin A concentrations significantly increased with disease worsening and lower RASS, and were related to bradykinin and substance P plasma concentrations. Also, orexin A significantly correlated with LOS in the ICU and death in COVID-19 patients. Additionally, we showed that plasma bradykinin, substance P, and orexin A increased with lower RASS, and this effect seemed to be independent of the number of anaesthetic drugs. Notably, the combined measurements of bradykinin, substance P, and orexin A in COVID-19 patients with RASS –3 to –5 separated survivors from non-survivors of COVID-19 in a PCA when categorised by age. These results suggest that plasma concentrations of orexin A play an important role in severely ill COVID-19 patients.

Interestingly, it has been shown earlier that in patients with systemic inflammation in the ICU, orexin A could not reflect the severity of illness.^39^ These results align with our findings, which revealed no significant association of orexin A with inflammatory markers. However, Ogawa and colleagues^40^ reported that peripherally administered orexin A improved survival in a murine model of sepsis by mainly acting in the medullary raphe nucleus through OX_2_R. A preliminary follow-up study by the same authors found reduced cerebrospinal fluid orexin concentrations in rats and patients with systemic inflammation, supporting the hypothesis that orexin treatment may enhance recovery from sepsis.^41^ This was recently further supported by Guo and colleagues,^42^ who showed that intranasal administration of moderate amounts of synthetically derived orexin A reduced mortality, diminished the expression of the proinflammatory factors interleukin 1 beta (IL-1β), and tumour necrosis factor alpha (TNF-α), ameliorated cognitive and emotional deficits, and attenuated cerebral oedema, blood–brain barrier disruption, and ultrastructural brain damage in mice with sepsis-associated encephalopathy. Furthermore, systemic administration of orexin A ameliorated experimental autoimmune encephalomyelitis in mice by diminishing neuroinflammation.^18^ These results support an important role for the orexinergic system in severe inflammatory-driven diseases.

Our results suggested that orexin A acted at least partially in concert with bradykinin and substance P. This was particularly noticeable in patients with RASS –3 to –5, indicating a neurophysiological connection. The interplay between bradykinin and substance P and their cognate signalling pathways may influence the activity of orexin neurons through direct interaction or indirect effects via other neurochemical systems.^29^, ^30^, ^31^^,^^33^, ^34^, ^35^^,^^38^

In this context, it is important to emphasise that sedation behaviour in patients with severe CARDS has been described as severely impaired at various centres.^25^^,^^43^ Even direct data on other viral-induced ARDS showed a significant increase in the need for sedatives and analgesics to achieve adequate treatment compliance.^24^ Previously published data indicated a significant discrepancy in processed electroencephalogram (pEEG) documentation between the level of sedation observed clinically by RASS owing to inter- and counter-breathing on the ventilator, repeated coughing, and the documented pEEG, including increased burst suppression rate.^44^ The increasing plasma concentrations of bradykinin, substance P, and particularly orexin A, which we found with more pronounced sedation, may explain these discrepancies, particularly as this seems independent of the number of anaesthetic drugs. Furthermore, plasma concentrations of orexin A fluctuated massively in COVID-19 patients upon ROS, NIV, MV, or ECMO and were unusually high 1–3 days after the start of MV. Thereby, orexin A-mediated wakefulness properties may blunt the effects of anaesthetics, potentially requiring higher doses to achieve an adequate level of sedation. Indeed, studies have shown that cerebrospinal fluid concentrations of orexin A are reduced during anaesthesia and that orexin A plays a crucial role to promote and maintain wakefulness during anaesthesia emergence.^10^^,^^38^^,^^45^, ^46^, ^47^, ^48^ It has been shown that the intracerebroventricular injection of orexin A in rats affected the duration of propofol anaesthesia, with an increase in orexin A concentrations accelerating the emergence from anaesthesia.^46^ Furthermore, orexins have been found to increase mean arterial blood pressure and heart rate when administered to urethane-anaesthetised rats, with orexin A being more effective in this regard than orexin B.^49^

There is a discrepancy between the clinical and EEG-based determination of sedation with RASS. In particular, pEEG indices are unable to reflect brain stem responses to noxious stimuli such as coughing on the tracheal tube. They are less reliable during discontinued EEG patterns, such as burst suppression or low-amplitude EEG. These patterns are commonly observed in patients with high concentrations of hypnotic drugs, where the term ‘sedation’ may be a misnomer. Consequently, it is imperative to conduct regular observation and documentation of the level of sedation using pEEG in cases of intended deep sedation in critical treatment, as evidenced by such observations.^50^ Nevertheless, the assessment of the overall sedation level and the adequate allocation of resources in cases of severe ARDS remains a challenge. The overuse of sedative drugs to suppress arousal responses to noxious stimuli may have adverse effects on the patient’s brain health.^26^

In this study, orexin A plasma concentrations differed slightly at the significance level between male and female patients and between patients with and without diabetes mellitus, cardiovascular disease, or respiratory disease. This may be a coincidence, as most patients in our cohort were male and a majority had more than one pre-existing disease.

The elevated plasma substance P concentrations in the 2.5-yr follow-up cohort were remarkable. This increase may be associated with the physical, cognitive, and neuropsychological impairments commonly seen in post-ICU and post-COVID syndromes. It has been recently shown that substance P was overexpressed in olfactory neurons and plays differential roles in those with persistent post-COVID-19 olfactory dysfunction.^51^

Several limitations need to be addressed. First, drug treatment may confound the results, as all COVID-19 ICU patients were on extensive medication. Second, the cross-sectional design of this study and the heterogenous study cohort cannot provide evidence of causality. Therefore, it is not possible to establish a genuine cause-and-effect relationship. Third, assessing RASS for ICU patients is highly subjective.

However, we report a novel link between orexin A, substance P, and bradykinin, which is associated with disease progression in COVID-19, LOS in the ICU, and the survival of severe COVID-19 patients. These findings may also have implications for other serious respiratory viral diseases, such as influenza or respiratory syncytial virus. Future studies are necessary to analyse the orexinergic system in severely ill, ventilated, and sedated patients to clarify the relationships mechanistically. Generalisation of other viral or inflammatory diseases will also be the focus of follow-up trials.

## Conclusions

We hypothesise that an imbalance of the bradykinin axis in severe COVID-19 influences substance P and orexin signalling, which ultimately affects disease worsening, ICU LOS, and survival of COVID-19 patients. Elevated substance P concentrations in the 2.5-yr follow-up cohort may be associated with physical, cognitive, and neuropsychological impairments commonly seen in post-ICU and post-COVID syndromes. In addition to COVID-19, orexin A, substance P, bradykinin, and DABK, and the complex interplay between the renin–angiotensin system and the orexinergic system may also have implications for other serious respiratory viral diseases, such as influenza or respiratory syncytial virus.

## Authors’ contributions

Study conception and design: UH

Acquisition of data: AF, AS, EA, FT, UH

Data interpretation: AF, ST, SZ, UH

Data analysis: AS, EA, ST, UH

Manuscript drafting: AF, UH

Critical revision of the manuscript for important intellectual content: all authors

Final approval of the manuscript version to be published: all authors

All authors agree to be accountable for all aspects of the work, thereby ensuring that questions related to the accuracy or integrity of any part of the work are appropriately investigated and resolved.

## Data availability

The datasets used, analysed, or both, during the current study are available from the corresponding author upon reasonable request.

## Declaration of interests

The Department of Anaesthesiology, Intensive Care Medicine & Pain Therapy of the University Hospital Frankfurt, Goethe University, received support from B. Braun Melsungen, CSL Behring, Fresenius Kabi, and Vifor Pharma for the implementation of Frankfurt‘s Patient Blood Management programme.

KZ has received honoraria for participation in advisory board meetings for Haemonetics and Vifor and received speaker fees from CSL Behring, Masimo, Pharmacosmos, Boston Scientific, Salus, iSEP, Edwards, and GE Healthcare. He is the Principal Investigator of the EU-Horizon 2020 project ENVISION (Intelligent plug-and-play digital tool for real-time surveillance of COVID-19 patients and smart decision-making in Intensive Care Units) and Horizon Europe 2021 project COVend (Biomarker and AI-supported FX06 therapy to prevent progression from mild and moderate to severe stages of COVID-19).

KZ leads as CEO the Christoph Lohfert Foundation and the Health, Patient Safety & PBM Foundation.

ANF received speaker fees from P.J. Dahlhausen & Co. GmbH, Cologne, Germany, and won the Sedana Medical Research Grant 2020 and the Thieme Teaching Award 2022.

The remaining authors declare no potential conflicts of interest.

## References

[1] Sakurai T. (2007). The neural circuit of orexin (hypocretin): maintaining sleep and wakefulness. Nat Rev Neurosci.

[2] Sakurai T. (2014). The role of orexin in motivated behaviours. Nat Rev Neurosci.

[3] Mogavero M.P., Silvani A., Lanza G., DelRosso L.M., Ferini-Strambi L., Ferri R. (2023). Targeting orexin receptors for the treatment of insomnia: from physiological mechanisms to current clinical evidence and recommendations. Nat Sci Sleep.

[4] Wang C., Wang Q., Ji B. (2018). The orexin/receptor system: molecular mechanism and therapeutic potential for neurological diseases. Front Mol Neurosci.

[5] Nishino S., Ripley B., Overeem S., Lammers G.J., Mignot E. (2000). Hypocretin (orexin) deficiency in human narcolepsy. Lancet.

[6] Chen Q., Lecea L de, Hu Z., Gao D. (2015). The hypocretin/orexin system: an increasingly important role in neuropsychiatry. Med Res Rev.

[7] LaCrosse A.L., Olive M.F. (2013). Neuropeptide systems and schizophrenia. CNS Neurol Disord Drug Targets.

[8] Tsuchimine S., Hattori K., Ota M. (2019). Reduced plasma orexin-A concentrations in patients with bipolar disorder. Neuropsychiatr Dis Treat.

[9] Yu H., Ni P., Zhao L. (2023). Decreased plasma neuropeptides in first-episode schizophrenia, bipolar disorder, major depressive disorder: associations with clinical symptoms and cognitive function. Front Psychiatry.

[10] Chemelli R.M., Willie J.T., Sinton C.M. (1999). Narcolepsy in orexin knockout mice: molecular genetics of sleep regulation. Cell.

[11] Lin L., Faraco J., Li R. (1999). The sleep disorder canine narcolepsy is caused by a mutation in the hypocretin (orexin) receptor 2 gene. Cell.

[12] Igarashi N., Tatsumi K., Nakamura A. (2003). Plasma orexin-A concentrations in obstructive sleep apnea-hypopnea syndrome. Chest.

[13] Nakabayashi M., Suzuki T., Takahashi K. (2003). Orexin-A expression in human peripheral tissues. Mol Cell Endocrinol.

[14] Korczynski W., Ceregrzyn M., Matyjek R. (2006). Central and local (enteric) action of orexins. J Physiol Pharmacol.

[15] Zhang S., Blache D., Vercoe P.E. (2005). Expression of orexin receptors in the brain and peripheral tissues of the male sheep. Regul Pept.

[16] Ye W., Yan Y., Tang Y. (2021). Orexin-A attenuates inflammatory responses in lipopolysaccharide-induced neural stem cells by regulating NF-KB and phosphorylation of MAPK/P38/Erk pathways. J Inflamm Res.

[17] Messal N., Fernandez N., Dayot S. (2018). Ectopic expression of OX1R in ulcerative colitis mediates anti-inflammatory effect of orexin-A. Biochim Biophys Acta Mol Basis Dis.

[18] Becquet L., Abad C., Leclercq M. (2019). Systemic administration of orexin A ameliorates established experimental autoimmune encephalomyelitis by diminishing neuroinflammation. J Neuroinflammation.

[19] Sun M., Wang W., Li Q., Yuan T., Weng W. (2018). Orexin A may suppress inflammatory response in fibroblast-like synoviocytes. Biomed Pharmacother.

[20] COVID-19 epidemiological update. Available from https://www.who.int/publications/m/item/covid-19-epidemiological-update-edition-170 (accessed 26 May 2025)

[21] COVID-ICU Group on behalf of the REVA Network and the COVID-ICU Investigators (2021). Clinical characteristics and day-90 outcomes of 4244 critically ill adults with COVID-19: a prospective cohort study. Intensive Care Med.

[22] Grasselli G., Zangrillo A., Zanella A. (2020). Baseline characteristics and outcomes of 1591 patients infected with SARS-CoV-2 admitted to ICUs of the Lombardy region, Italy. JAMA.

[23] Flinspach A.N., Booke H., Zacharowski K., Balaban Ü., Herrmann E., Adam E.H. (2021). High sedation needs of critically ill COVID-19 ARDS patients-a monocentric observational study. PLoS One.

[24] Wongtangman K., Santer P., Wachtendorf L.J. (2021). Association of sedation, coma, and in-hospital mortality in mechanically ventilated patients with coronavirus disease 2019-related acute respiratory distress syndrome: a retrospective cohort study. Crit Care Med.

[25] Flinspach A.N., Booke H., Zacharowski K., Balaban Ü., Herrmann E., Adam E.H. (2022). Associated factors of high sedative requirements within patients with moderate to severe COVID-19 ARDS. J Clin Med.

[26] Chang B.A., Cassim T.Z., Mittel A.M., Brambrink A.M., García P.S. (2023). Frontal electroencephalography findings in critically ill COVID-19 patients. J Neurosurg Anesthesiol.

[27] Formenti P., Bichi F., Castagna V., Pozzi T., Chiumello D. (2021). Nutrition support in patients with acute respiratory distress syndrome COVID-19. Nutr Clin Pract.

[28] Zinn S., Talbot S.R., Rajapakse D. (2023). Evidence from fatal COVID-19 for targeting the bradykinin metabolism-a single-center cohort study. Shock.

[29] Nakajima Y., Nakajima S. (2010). Measurement of orexin (hypocretin) and substance P effects on constitutively active inward rectifier K(+) channels in brain neurons. Methods Enzymol.

[30] Iversen L.L., Jessell T., Kanazawa I. (1976). Release and metabolism of substance P in rat hypothalamus. Nature.

[31] Ogata N., Abe H. (1982). Neuropharmacology in the brain slice: effects of substance P on neurons in the guinea-pig hypothalamus. Comp Biochem Physiol C Comp Pharmacol.

[32] Kanazawa I., Emson P.C., Cuello A.C. (1977). Evidence for the existence of substance P-containing fibres in striato-nigral and pallido-nigral pathways in rat brain. Brain Res.

[33] Kang X., Tang H., Liu Y., Yuan Y., Wang M. (2021). Research progress on the mechanism of orexin in pain regulation in different brain regions. Open Life Sci.

[34] Mukerjee S., Lazartigues E. (2018). Sympathetic nerve activity and neuro-inflammation: Who is in the driver's seat?. Acta Physiol (Oxf).

[35] Watanabe S., Kuwaki T., Yanagisawa M., Fukuda Y., Shimoyama M. (2005). Persistent pain and stress activate pain-inhibitory orexin pathways. Neuroreport.

[36] World Medical Association (2013). World Medical Association Declaration of Helsinki: ethical principles for medical research involving human subjects. JAMA.

[37] Cohen J. (2013).

[38] Kelz M.B., García P.S., Mashour G.A., Solt K. (2019). Escape from oblivion: neural mechanisms of emergence from general anesthesia. Anesth Analg.

[39] Akaishi M., Hashiba E., Takekawa D., Kushikata T., Hirota K. (2022). Plasma orexin A does not reflect severity of illness in the intensive care units patients with systemic inflammation. JA Clin Rep.

[40] Ogawa Y., Irukayama-Tomobe Y., Murakoshi N. (2016). Peripherally administered orexin improves survival of mice with endotoxin shock. Elife.

[41] Ogawa Y., Shimojo N., Ishii A., Tamaoka A., Kawano S., Inoue Y. (2022). Reduced CSF orexin concentrations in rats and patients with systemic inflammation: a preliminary study. BMC Res Notes.

[42] Guo J., Kong Z., Yang S. (2024). Therapeutic effects of orexin-A in sepsis-associated encephalopathy in mice. J Neuroinflammation.

[43] Sigala M.I., Dreucean D., Harris J.E. (2023). Comparison of sedation and analgesia requirements in patients with SARS-CoV-2 versus non-SARS-CoV-2 acute respiratory distress syndrome on veno-venous ECMO. Ann Pharmacother.

[44] Flinspach A.N., Zinn S., Zacharowski K., Balaban Ü., Herrmann E., Adam E.H. (2022). Electroencephalogram-based evaluation of impaired sedation in patients with moderate to severe COVID-19 ARDS. J Clin Med.

[45] Kushikata T., Hirota K., Yoshida H. (2003). Orexinergic neurons and barbiturate anesthesia. Neuroscience.

[46] Shirasaka T., Yonaha T., Onizuka S., Tsuneyoshi I. (2011). Effects of orexin-A on propofol anesthesia in rats. J Anesth.

[47] Zhang L.-N., Yang C., Ouyang P.-R. (2016). Orexin-A facilitates emergence of the rat from isoflurane anesthesia via mediation of the basal forebrain. Neuropeptides.

[48] Kushikata T., Yoshida H., Kudo M., Kudo T., Hirota K. (2010). Changes in plasma orexin A during propofol-fentanyl anaesthesia in patients undergoing eye surgery. Br J Anaesth.

[49] Chen C.T., Hwang L.L., Chang J.K., Dun N.J. (2000). Pressor effects of orexins injected intracisternally and to rostral ventrolateral medulla of anesthetized rats. Am J Physiol Regul Integr Comp Physiol.

[50] Rasulo F.A., Hopkins P., Lobo F.A. (2023). Processed electroencephalogram-based monitoring to guide sedation in critically ill adult patients: recommendations from an international expert panel-based consensus. Neurocrit Care.

[51] Schirinzi T., Lattanzi R., Maftei D. (2023). Substance P and Prokineticin-2 are overexpressed in olfactory neurons and play differential roles in persons with persistent post-COVID-19 olfactory dysfunction. Brain Behav Immun.

