# Supplementary material for: Systemic role of orexin A, substance P, bradykinin, and DABK in severe COVID-19 and 2.5-yr follow-ups: an observational study
Source: BJA Open. 2025 Jun 5;14:100415. doi: 10.1016/j.bjao.2025.100415 (PMC12173137; doi:10.1016/j.bjao.2025.100415)
Supplement: Supplementary file 1 [file mmc1.docx]

**Supplement**

**The systemic role of Orexin A, Substance P, Bradykinin and DABK in severe COVID-19 and 2.5-year follow-ups: an observational study**

Ulrike Heinicke^1*^, Steven R Talbot^2^, Filippos Thanasis^1^, Elisabeth H Adam^1^, Andreas von Knethen^1^, Andrea U. Steinbicker^1^, Sebastian Zinn^1^, Kai Zacharowski^1,3^, Armin N. Flinspach^1#^

^1^Goethe University, University Hospital Frankfurt, Department of Anaesthesiology, Intensive Care Medicine and Pain Therapy, 60590 Frankfurt am Main, Germany

^2^Institute for Laboratory Animal Science, Hannover Medical School, 30625 Hannover, Germany

^3^Fraunhofer – Institute for Translational Medicine and Pharmacology (ITMP), 60596 Frankfurt am Main Frankfurt am Main, Germany

Correspondence:

Ulrike Heinicke

Goethe University

University Hospital Frankfurt

Department of Anaesthesiology, Intensive Care Medicine and Pain Therapy

Theodor-Stern-Kai 7

60590 Frankfurt am Main

Germany

Email: [heinicke@med.uni-frankfurt.de](mailto:heinicke@med.uni-frankfurt.de)

**Running title:** Orexin A, Substance P, and Bradykinin in COVID-19: A 2.5-Year Study

**Supplement figure legends**

**Supplementary Figure S1**

A violin plot of plasma orexin A in females (n=20) and males (n=58) with COVID-19 is shown (A). The median of 14 healthy controls is presented as a dotted line. Statistical differences between the groups were assessed using the Kruskal-Wallis test. Plasma orexin A levels were plotted against age (B), BMI (C), and glucose levels (D). Statistical differences were assessed using Spearman’s correlation test. Trend analysis was performed with linear regression. Mean and errors (dotted lines) are shown.

**Supplementary Figure S2**

Violin plots of plasma orexin A in COVID-19 patients with (n=44) or without (n=34) CVD (A), with (n=17) or without (n=61) obesity (B), with (n=14) or without (n=64) resp. diseases (C) or with (n=23) or without (n=55) DM (D) are shown. The median of 14 healthy controls is presented as a dotted line. Statistical differences between the groups were assessed using the Kruskal-Wallis test. *p ˂ 0.05. Plasma orexin A levels were plotted against insulin levels (E). Statistical differences were assessed using Spearman’s correlation test. Trend analysis was performed with linear regression. Mean and errors (dotted lines) are shown.

CVD, cardiovascular diseases; DM, diabetes mellitus; resp., respiratory

**Supplementary Figure S3**

Plasma levels of bradykinin (A), substance P (B), or DABK (C) were plotted against p_a_ F_i_O_2_^-1^ ratio. Statistical differences were assessed using Spearman’s correlation test. Trend analysis was performed with linear regression. Mean and errors (dotted lines) are shown. *p ˂ 0.05, **p ˂ 0.01, ***p ˂ 0.001.

DABK, Des-Arg^9^-bradykinin

**Supplementary Figure S4**

Log_10_ plasma levels of bradykinin (A), substance P (B), or DABK (C), depending on disease progression, are depicted as box plots. Patients of the COVID-19 cohort and 2.5-year follow-ups were divided into eight groups resembling the clinical course of COVID-19. Patients requiring oxygen supplementation (ROS) at the beginning of the disease were divided into group 0, patients 0-3 days before connection to MV into group 1, patients 1-3 days after connection to MV into group 2, patients 8-10 days after connection to MV into group 3, patients with implemented ECMO into group 4, patients were plasma samples were collected on the day of death into group 5, patients were plasma samples were collected on the day of extubation or discharge into group 6, and 2.5-year follow-ups of COVID-19 into group 7. Statistical differences were assessed using the Kruskal-Wallis test; *p ˂ 0.05, **p ˂ 0.01, ***p ˂ 0.001.

Mean and standard deviation of Log_10_ plasma levels of bradykinin, substance P, DABK, or orexin A according to group classification of COVID-19 disease progression are shown (D). Box plots of DABK plasma levels depending on RASS are shown (E). Statistical differences were assessed using the Kruskal-Wallis test; *p ˂ 0.05, **p ˂ 0.01, ***p ˂ 0.001.

ECMO, extracorporeal membrane oxygenation; MV, mechanical ventilation; RASS, Richmond Agitation-Sedation Scale; ROS, requirement for oxygen supplementation.

**Supplementary Figure S5**

Log_10_ plasma levels of bradykinin (A), substance P (B), or orexin A (C) are plotted against the number (No. of anaesthetics). COVID-19 patients with RASS -3 to -5 are depicted in yellow, orange, or red circles, respectively. The median of 14 healthy controls is presented as a dotted line. Statistical differences were assessed using ANOVA.
