# Supplementary material for: Systemic role of orexin A, substance P, bradykinin, and DABK in severe COVID-19 and 2.5-yr follow-ups: an observational study
Source: BJA Open. 2025 Jun 5;14:100415. doi: 10.1016/j.bjao.2025.100415 (PMC12173137; doi:10.1016/j.bjao.2025.100415)
Supplement: Supplementary file 2 [file mmc2.pptx]

## Slide 1
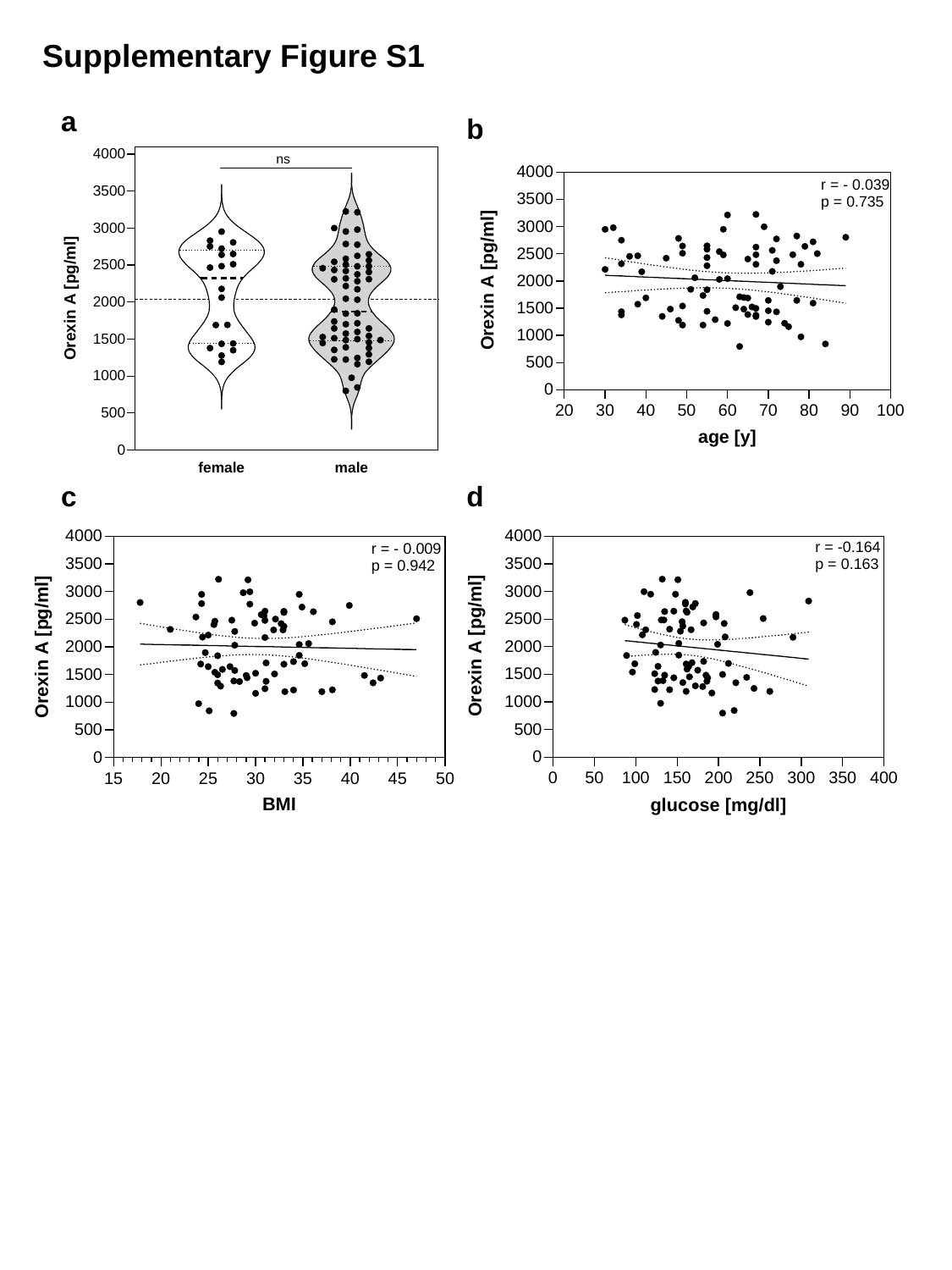

Supplementary Figure S1
a
b
c
d

## Slide 2
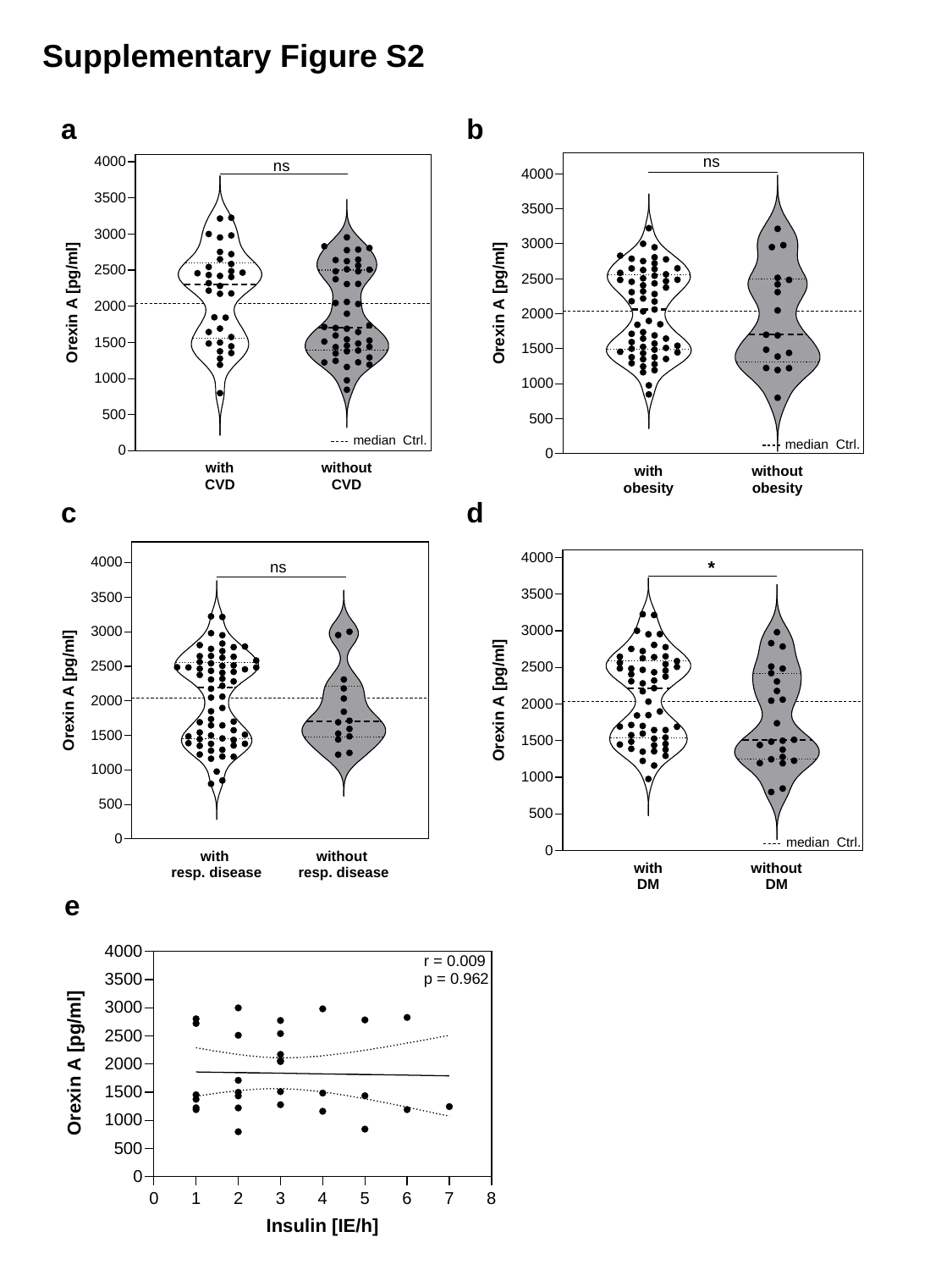

Supplementary Figure S2
a
b
c
d
e

## Slide 3
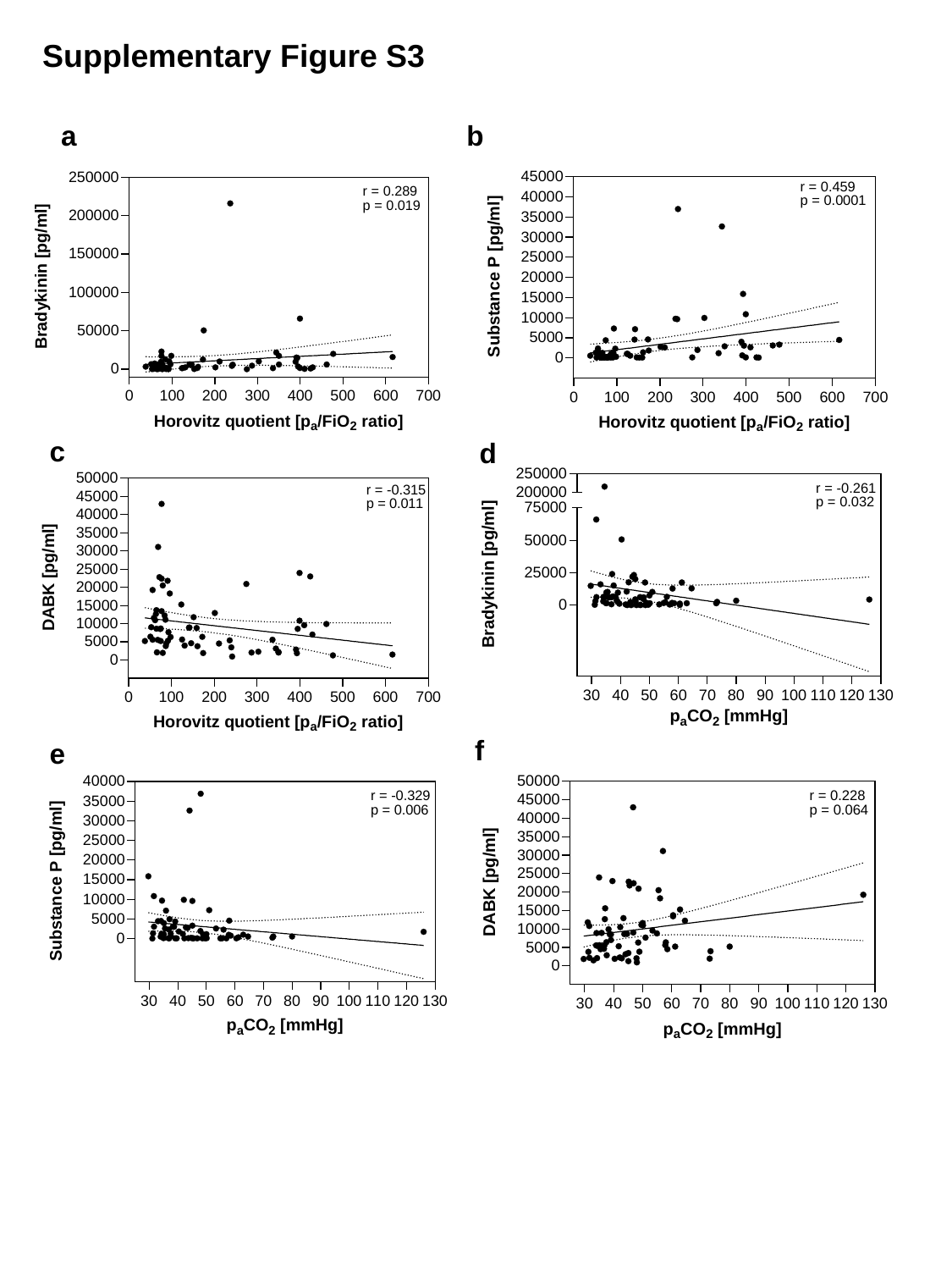

Supplementary Figure S3
a
b
c
d
f
e

## Slide 4
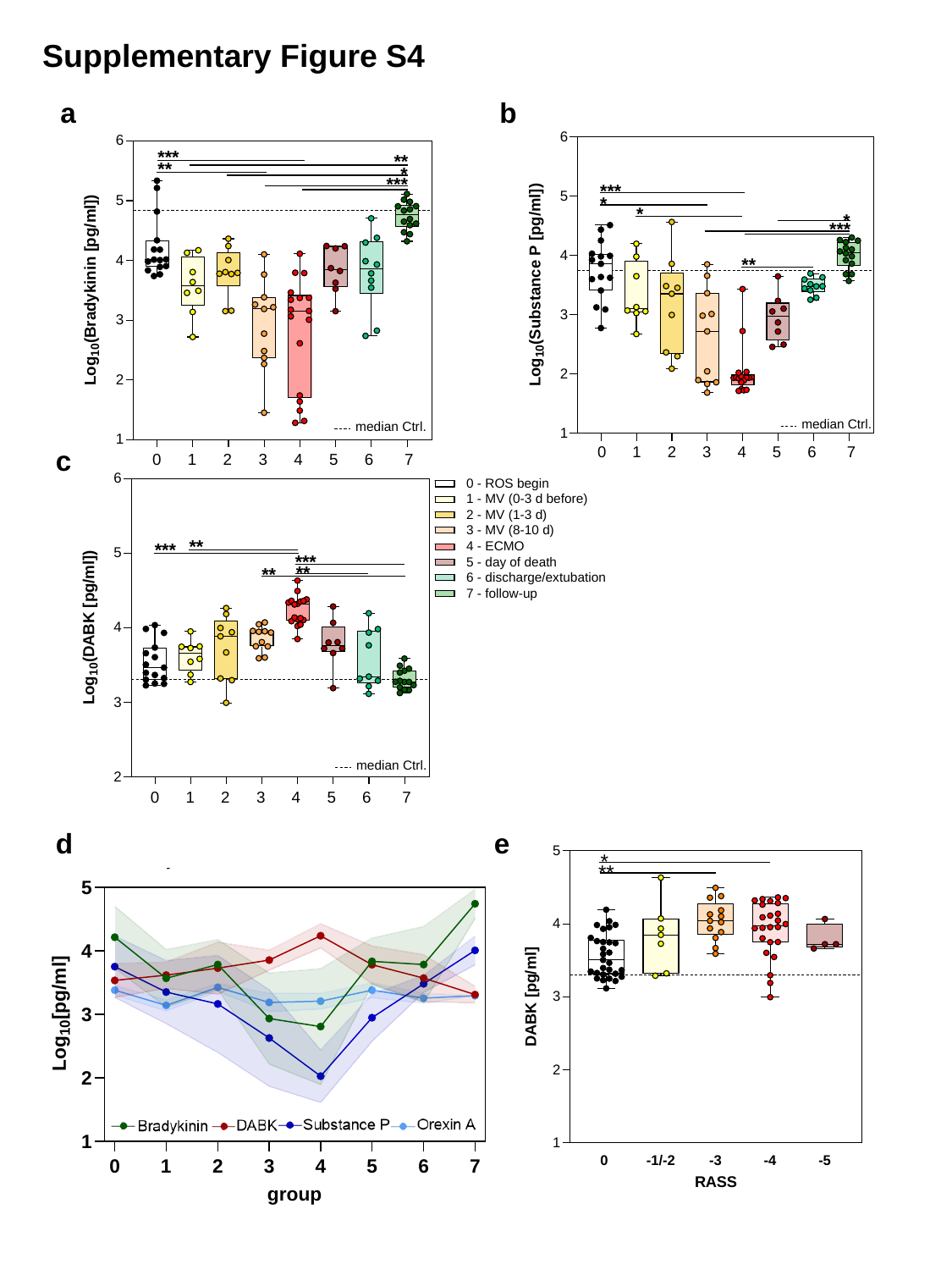

Supplementary Figure S4
a
b
c
d
e

## Slide 5
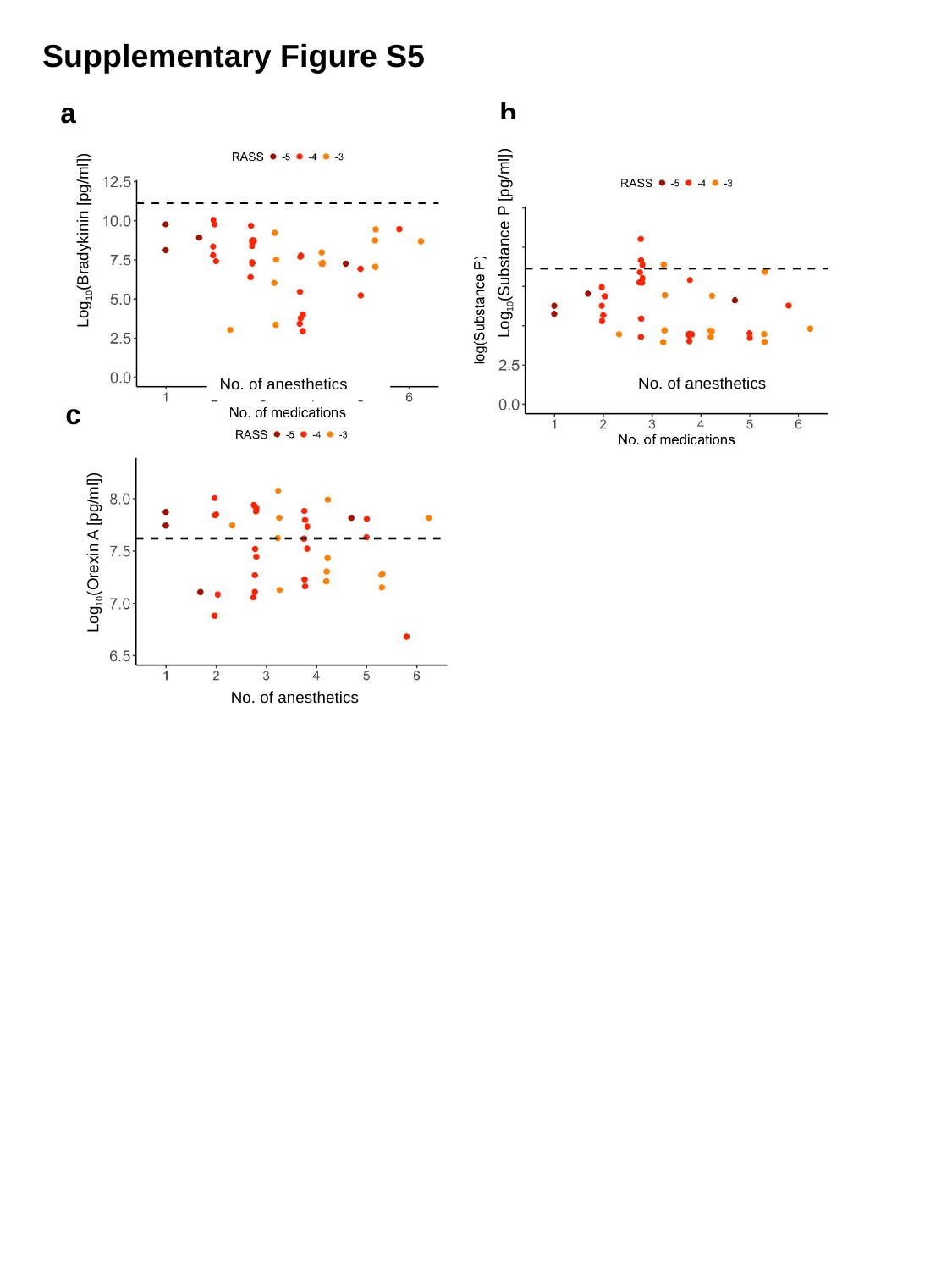

Supplementary Figure S5
a
b
Log10(Bradykinin [pg/ml])
Log10(Substance P [pg/ml])
No. of anesthetics
No. of anesthetics
c
Log10(Orexin A [pg/ml])
No. of anesthetics
